# Migration background and juvenile mental health: a descriptive retrospective analysis of diagnostic rates of psychiatric disorders in young people

**DOI:** 10.3402/gha.v6i0.20187

**Published:** 2013-06-19

**Authors:** Tilman Jakob Gaber, Samira Bouyrakhen, Beate Herpertz-Dahlmann, Ulrich Hagenah, Martin Holtmann, Christine Margarete Freitag, Lars Wöckel, Fritz Poustka, Florian Daniel Zepf

**Affiliations:** 1 Clinic for Child and Adolescent Psychiatry, Psychosomatics and Psychotherapy, RWTH Aachen University, Aachen, Germany; 2 JARA Translational Brain Medicine, Aachen and Jülich, Germany; 3 Department of Child and Adolescent Psychiatry, Psychosomatics and Psychotherapy, J.W. Goethe University, Frankfurt am Main, Germany; 4 Department of Child and Adolescent Psychiatry, Ruhr University Bochum, Bochum, Germany; 5 Jülich Research Centre, Institute for Neuroscience and Medicine, Jülich, Germany

**Keywords:** immigration issues, risk factors, mental health, children, adolescents

## Abstract

**Introduction:**

This article presents diagnostic rates for specific mental disorders in a German pediatric inpatient population over a period of 20 years with respect to migration background and socioeconomic status (SES).

**Methods:**

Diagnostic data were obtained over a period of 20 years from 8,904 patients who visited a child and adolescent psychiatry mental health service in Germany. Data from 5,985 diagnosed patients (ICD-9 and ICD-10 criteria) were included with respect to gender, migration background, and SES.

**Results:**

Migration- and gender-specific effects were found for both periods of assessment. The group of boys with a migration background showed significantly higher rates of reactions to severe stress, adjustment disorders, and posttraumatic stress disorder compared to their male, non-migrant counterparts. Conversely, boys without a migration background showed a significantly higher percentage rate of hyperkinetic disorders than male migrants. Similar results were found for female migrants in the latter assessment period (ICD-10). In addition, female migrants showed lower rates of emotional disorders whose onset occurs in childhood compared to their non-migrant counterparts.

**Conclusions:**

Data from this investigation provide preliminary evidence that the prevalence of various psychiatric disorders in children and adolescents is influenced by migration background and SES.

Migration and change of residence are two phenomena that are inherent to the cultural and social changes occurring in our modern industrialized world. Though both phenomena have occurred in many nations and throughout history, the ongoing globalization process has significantly contributed to increased migration movements around the globe (1). The underlying motivations of relocating to a foreign country are heterogeneous and include reasons related to education, employment, finances, natural disasters, and personal or political issues (2–11). The migration process itself is not only characterized by a broad variety of motivations, but there are also many heterogeneous factors that altogether contribute to the immigrants’ situations in their new environment. Thus, migration processes are reflected on the individual level, pose a risk to subjective health, and may result in physical and mental health problems. In particular, mental health can be significantly affected by a number of factors associated with migration. These factors relate to the country of origin, for example nutrition and lifestyle, and to the new country of residence, and they are relevant to mental and physical health in association with the experience of being a migrant (1, 11–16). It is not uncommon for migrants to experience discrimination, work- or school-related stressors and improved or worsened access to health care services. In particular, children and adolescents can be greatly affected by the migration process (17, 18). However, the variety of experiences and the reasons for migration indicate that not all children and adolescents with a migration background face similar experiences before or after migration. Apparent postmigration stressors include the loss of friendships, family and lifestyle, in addition to significant changes in social support, discrimination, language and cultural difficulties, and academic challenges (19). For example, recent data from Greece indicate that members of immigrant families had significantly lower job status, worse housing and economic situations and higher rates of non-insurance than non-immigrant families (18, 20). Further data from the United States reveal that immigrants had a lower risk of psychiatric disorders than natives prior to arrival in the United States but showed a trend toward the equalization of risk as the migrants resided longer in the host country (21).

As for Germany, the contextual situation remains somewhat ambiguous, and the findings are rather scarce and heterogeneous. An evaluation of the required general health examination prior to school entry revealed elevated prevalence rates for overweight and obesity in children with non-German nationality compared with the rates for German children (22–24). However, a recent investigation found that adolescent migrants showed no differences in physical health compared to non-migrants and reported higher anxiety and depression levels (25). Over the course of more than two decades, a number of studies have indicated elevated prevalence rates of juvenile psychiatric conditions for children and adolescents with a migration background in Germany (26–29), while others have found lower prevalence rates for those individuals compared to German children (30). There is some evidence that these differences are related to the specific country of origin (27, 31). For instance, children with a Greek or Italian background showed lower prevalence rates for psychiatric disorders, while higher rates were found for children with a Turkish background (27, 29, 30). One recent comprehensive nationwide study on the health-related quality of life in Germany indicates significant effects of age, gender, socioeconomic status (SES), and migration background, whereby younger age, low SES, and migration background were associated with increased psychopathology (32–34). Of note, migration-related mental health issues may be reflected differently in males and females; however, data from children and adolescents with a migration background in regard to potential gender differences are scarce (25, 35).

Because Germany has been an important immigration country for several decades, there are new challenges for health services to address with respect to the needs of a growing migrant population. This challenge is in fact highly relevant for other countries to which people immigrate or regions such as the United States, Canada, Australia, and the European Union. To the authors’ knowledge, no German studies have investigated possible differences in the frequency with which psychiatric disorders in children and adolescents with migration histories are diagnosed compared to those of non-migrants. This study provides a retrospective analysis of the frequency with which psychiatric disorders have been diagnosed using a large population of young patients with and without a migration history who sought mental health services in Germany.

## Methods

### Study sample

The study was approved by the Ethics Committee of the Faculty of Medicine at J.W. Goethe University of Frankfurt am Main, Germany, and was conducted in accordance with the Helsinki Declaration. This study comprises a retrospective analysis. Written and informed consent from the patients, caretakers, or guardians on behalf of the minor/child participants involved in the study for their information to be stored in the hospital database and used for research was not needed because the obtained data were collected routinely in the course of clinical care. The responsible ethics committee assessed the study protocol, approved this consent protocol, and provided a waiver to retrospectively analyze these routine data and to publish the results anonymously.

**Fig. 1:**
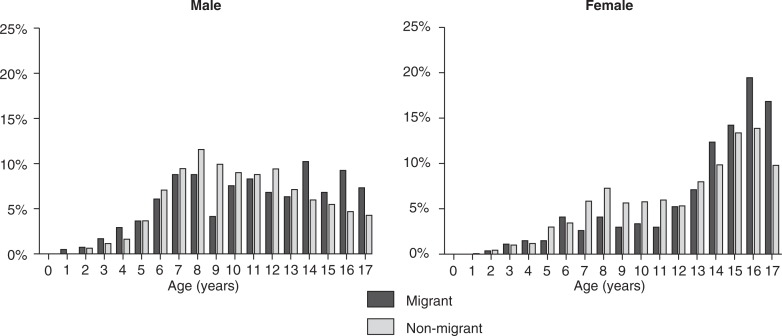
Average age distribution for male and female patients according to migration status.

The overall sample consisted of 8,904 subjects who were either inpatients or outpatients at a major mental health service. The analysis period was from 1988 to 2007 and consisted of two diagnostic periods (10 years each) in which two subsequent classification systems (ICD-9 and ICD-10) were applied. Two classification systems were used, and the data were stored and analyzed separately because the diagnostic criteria for some psychiatric disorders changed during the shift from the ICD-9 to ICD-10. Only patients who received at least one psychiatric diagnosis and were 17 years of age and under were included. If there were multiple periods of treatment, only the last period was used for statistical analyses. For reasons of limited data availability on SES, a total of 5,985 complete cases remained from the initial data set. The proportion of migrants and non-migrants in the final sample (22.6 vs. 77.4%) was similar to that in the initial data set (22.2 vs. 77.8%) and to the sociodemographic characteristics of the hospital's catchment area (approximately 20 vs. 80%). Notably, similar proportions for adults with a migration background were recently reported among psychiatric inpatients for a representative German survey (31). More boys than girls (64.6 vs. 35.4%) were diagnosed during the period of assessment and thus entered the final sample (64.5 vs. 35.5%). Notably, the age distribution of migrant and non-migrant patients in the study sample (Fig. 1) showed a statistically distinct pattern for all comparison groups, such as gender and classification system (*p*<0.01). The complete characteristics of the study sample and respective subgroups are given in Table 1.

**Table 1:** The characteristics of the two study groups, which consisted of patients with and without a migration background (number of patients [*n*] and percentage of sample, age±standard deviation [SD], parental socioeconomic status) for two periods of assessment

		Migrants			Non-migrants			Migrants vs. non-migrants *p*_adj_ (χ^2^)			
				
		Total	Males	Females	Total	Males	Females	Males	Females		
*ICD-9 assessment period*								0.074 (3.191)α			
	*n*	652	408	244	1,883	1,251	635				
	%		62.6	37.4		66.4	33.6				
Parental socioeconomic status								0.000 (74.9)	0.000 (103.6)		
Low	%		27.0	38.5		10.0	9.4				
Mid-low	%		64.7	54.9		76.7	77.3				
Mid-high	%		5.9	4.9		9.0	8.7				
High	%		2.5	1.6		4.2	*4*.*1*				
Age±SD	Years							0.000 (41.7)	0.000 (42.5)		
0–2	%		2.0	0.6		0.9	0.7				
3–5	%		6.4	3.1		8.1	8.1				
6–9	%		32.5	14.1		37.8	24.5				
10–13	%		30.5	18.4		34.9	25.6				
14–17	%		28.5	63.8		18.3	41.1				
*ICD-10 assessment period*								0.002 (10.0)α	
	*n*	702	412	290	2,748	1,789	959				
	%		58.7	41.3		65.1	34.9				
Parental socioeconomic status								0.000 (182.6)	0.000 (88.3)		
Low	%		47.1	42.1		17.2	17				
Mid-low	%		38.3	40		60.1	61.1				
Mid-high	%		6.8	6.9		16.4	13.3				
High	%		7.8	11		6.3	*8*.*6*				
Age±SD	Years							0.000 (52.2)	0.005 (14.8)		
0–2	%		1.9	0.0		0.7	0.3				
3–5	%		9.5	4.8		7.0	5.6				
6–9	%		23.4	14.0		35.6	21.5				
10–13	%		27.2	19.3		33.8	25.5				
14–17	%		38.0	61.8		22.9	47.0				

The diagnosis was obtained in accordance with either the ICD-9 or ICD-10 criteria for an assessment period, with each period of assessment lasting 10 years.

α These values refer to the proportion of male and female patients in migrants vs. non-migrants.

### Data assessment

Diagnostic and demographic data were taken from the KJP-BADO computerized documentation database (ZYRES Digital Media Systems, Frankfurt am Main, Germany) used at the aforementioned mental health service. KJP-BADO allows for the collection and administration of data in child and adolescent psychiatry and is widely used throughout German-speaking countries as an assessment and documentation system for clinical and research data. The migration background was assessed using citizenship data and included foreign-born and Germany-born youths. SES was assessed based on the parents’ educational and occupational achievements in accordance with a commonly used and established standardized procedure (36).

### Data analysis

Data analysis was performed using the SPSS (version 17) software package (SPSS, Chicago, IL, USA). Cross-tabulations were used to calculate the diagnostic frequencies of each category of disorders among children and juveniles with and without a migration background. Assessment periods (ICD-9 and ICD-10) and gender were analyzed individually. Adjusted odds ratios were used to examine associations between comparison groups and each specific psychiatric disorder. Standard errors and 95% confidence limits were estimated. To control for potential socioeconomic bias, stratified Cochran–Mantel–Haenszel statistics were applied. Incorporating parents’ SES as a control variable reduced the sample size by 17.9% due to missing SES data. Nevertheless, the missing data rate did not differ significantly between comparison groups (*p*=0.358, χ^2^=0.957) nor did the final sample differ significantly from the initial data with respect to gender (*p*=0.884, χ^2^=0.021) or citizenship (*p*=0.494, χ^2^=0.467). Some patients met criteria for more than one diagnostic category; indeed, 14.1% met criteria for two categories, while 1.2% met criteria for three or more categories. However, the proportion of patients with multiple diagnoses did not differ significantly between the comparison groups (ICD-9: *p*=0.686, χ^2^=0.142; ICD-10: *p =*0.082, χ^2^=3.295). Significant *p*-values were subjected to α-adjustment according to the Bonferroni–Holm procedure (37). The adjusted *p*-values are indicated by *p*_adj_. The level of statistic significance was *p*_adj_=0.05. Statistical inferences were only performed for those data surpassing a minimal cell frequency of 10 cases per category.

## Results

### Demographic comparisons

Overall, the migrant and non-migrant patient groups differed significantly with respect to SES (*p*=0.000, χ^2^=32.847). The parents of migrant patients were less likely to have reached a higher educational and occupational level compared to the parents of non-migrant patients (12.0 vs. 18.6% [OR = 1.69, CI (95%) = 1.41–2.02]). The proportion of patients with multiple diagnoses did not differ significantly between comparison groups (ICD-9: *p*=0.686, χ^2^=0.142; ICD-10: *p =*0.082, χ^2^=3.295). For assessment period I (ICD-9), there was no significant difference in the sex ratio between patients with and without a migration background (37.4 vs. 33.6%; *p*=0.074, χ^2^=3.191). In the latter period (ICD-10), the proportion of female patients with a migration background was significantly higher than patients without a migration background (41.3 vs. 34.9%; *p*=0.002, χ^2^=10.0). Of note, patients with a migration background were more likely to be outpatients than inpatients or day-care patients than were non-migrants (ICD-9 and ICD-10 assessment period together: 65.58 vs. 34.42%; *p*=0.000; χ^2^=14.938).

### Disorders with higher diagnostic rates in migrants

**Fig. 2:**
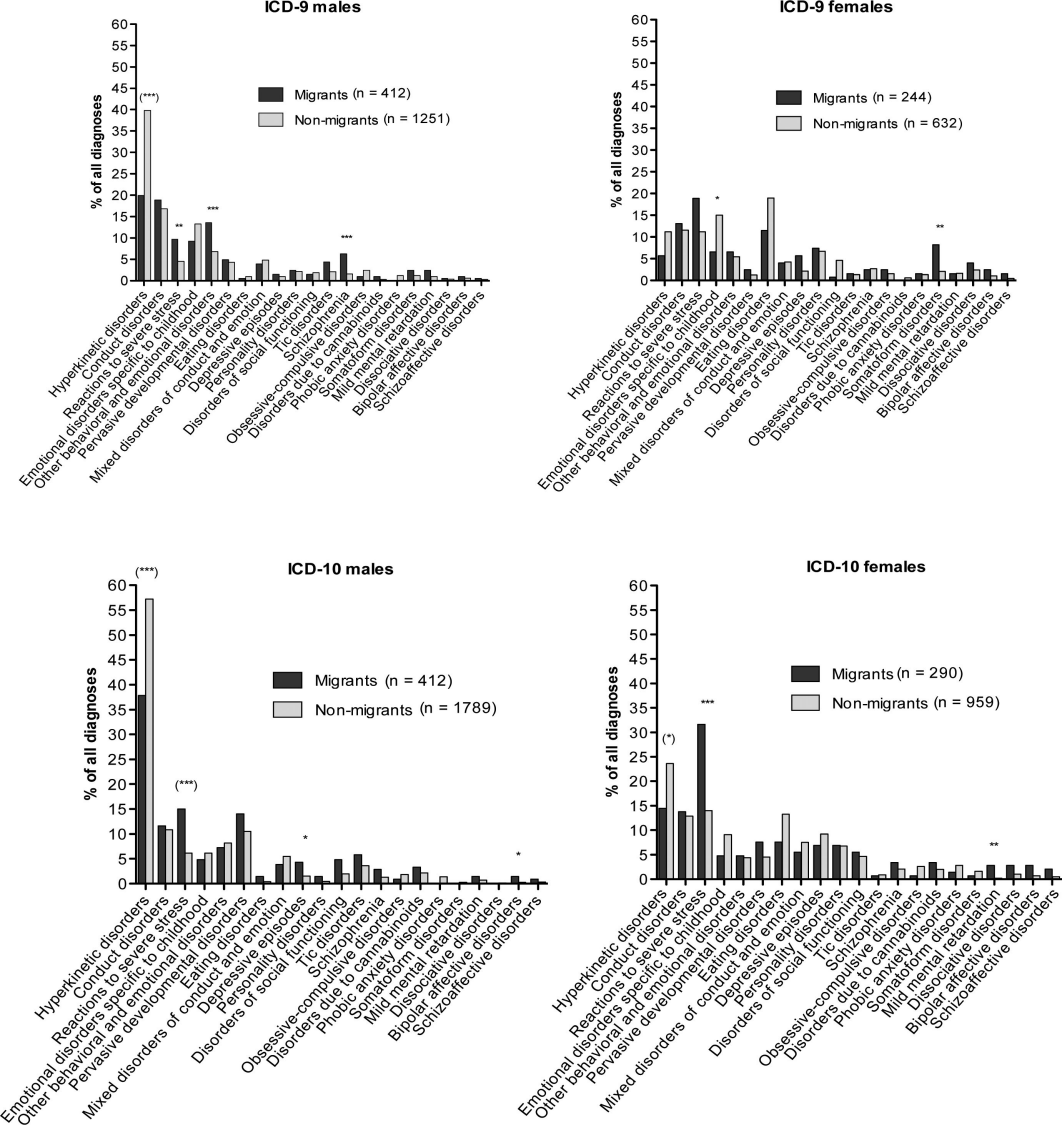
Distribution of disorders for the ICD-9 and ICD-10 assessment periods. Data are presented for the total sample, for each group (migrants vs. non-migrants) and by gender (*n*=number of patients,%). Asterisks indicate the level of significance after α-adjustment (*p_adj_<0.05; **p_adj_<0.01; ***p_adj_<0.001). Parentheses indicate a violation of the strata homogeneity prerequisite of the Cochran–Mantel–Haenszel procedure.

We detected higher diagnostic rates for reactions to severe stress and adjustment disorders in male migrants for both assessment periods (ICD-9 and ICD-10; Tables 2 and 4). This relationship was equally significant for females during the latter diagnostic period (ICD-10), but only at pre-adjusted significance levels with respect to diagnostic period I (ICD-9; *p*=0.005, χ^2^=7.881, Tables 3 and 5). Substantial interaction effects for SES were found for male patients during the ICD-10 period for the outlined findings (*p*=0.000, χ^2^=19.919, Fig. 2).

Depressive episodes were found at higher rates during the ICD-10 period in male migrants but not in female migrants, whereas for the ICD-9, this relationship was initially significant in females but did not surpass the adjusted level of significance (*p*=0.012, χ^2^=6.316; Tables 2, 3, 4 and 5). Male migrants had higher rates of behavioral and emotional disorders than male non-migrants, but only during the ICD-9 assessment period. There were higher rates for bipolar affective disorders in male migrants, but only for the ICD-10 assessment period. Migration background in females had no significant effect on the presence of bipolar affective disorders measured in either ICD-9 or ICD-10 (adjusted and unadjusted *p*-values). Moreover, there were no interactions with SES regarding these findings, as related to the diagnoses mentioned (depressive episodes, behavioral and emotional disorders, bipolar affective disorders).

**Table 2:** Comparison of disorders for the ICD-9 assessment period in male subjects

	Migrants	Non-migrants				
	
Disorders (ICD-9)	(*n =*408)	(*n =*1251)	χ^2^_CMH_	*p*	*p*_adj_	OR_MH_
Hyperkinetic disorders	20.1	39.8	51.3*	0.0000	0.0000	0.4 (0.3–0.5)
Conduct disorders	19.1	16.8	0.82	n.s.	n.s.	1.2 (0.9–1.5)
Reactions to severe stress	9.8	4.5	15.61	0.0001	0.0014	2.3 (1.5–3.6)
Emotional disorders specific to childhood	9.3	13.3	3.88	0.0490	0.7352	0.7 (0.5–1.0)
Other behavioral and emotional disorders**	4.9	6.8	16.48	0.0000	0.0009	2.1 (1.5–3.0)
Pervasive developmental disorders	13.7	4.3	0.24	n.s.	n.s.	1.2 (0.7–2.0)
Eating disorders	3.9	1.0	0.23	n.s.	n.s.	0.5 (0.1–2.5)
Mixed disorders of conduct and emotion	0.5	4.8	n.a.	n.a.	n.a.	n.a.
Depressive episodes	2.5	1.0	0.34	n.s.	n.s.	1.5 (0.6–4.2)
Personality disorders	1.5	2.2	0.01	n.s.	n.s.	1.1 (0.5–2.3)
Disorders of social functioning	1.5	1.9	0.13	n.s.	n.s.	0.8 (0.3–1.9)
Tic disorders	4.4	2.1	5.58	0.0182	0.3088	2.2 (1.2–4.0)
Schizophrenia	6.4	1.6	23.28	0.0000	0.0000	4.2 (2.3–7.6)
Obsessive–compulsive disorders	1.0	2.4	n.a.	n.a.	n.a.	n.a.
Disorders due to cannabinoids	1.0	0.3	n.a.	n.a.	n.a.	n.a.
Phobic anxiety disorders	0.0	1.2	n.a.	n.a.	n.a.	n.a.
Somatoform disorders	2.5	1.2	2.47	n.s.	n.s.	2.1 (0.9–4.8)
Mild mental retardation	2.5	1.0	4.02	0.0449	0.7181	2.5 (1.1–5.8)
Dissociative disorders	0.5	0.4	n.a.	n.a.	n.a.	1.2 (0.2–6.5)
Bipolar affective disorders	1.0	0.6	n.a.	n.a.	n.a.	1.7 (0.5–6.1)
Schizoaffective disorders	0.5	0.3	n.a.	n.a.	n.a.	1.7 (0.3–10.0)

Data are presented for the total sample for each group (migrants vs. non-migrants) for male subjects (*n =*number of patients,%). Statistical comparisons for each disorder were performed using the SES-adjusted chi-square (χ^2^) comparisons. Significant *p*-values underwent α-adjustment according to the Bonferroni–Holm procedure with adjusted *p*-values indicated by *p*_adj_, while the level of statistical significance remained at *p <*0.05 (n.a.=not applicable due to low cell frequencies, n.s.=not significant). Data are presented in descending order with respect to the diagnostic frequency of the respective disorders in the total sample. Shaded values represent adjusted and unadjusted significant findings that were also detected for the ICD-10 period of assessment.

* = Violation of the strata homogeneity prerequisite of the Cochran–Mantel–Haenszel procedure (see text for details).

** = A significant relationship was also found after α-adjustment in females for the ICD-9 period of assessment.

**Table 3:** Comparison of disorders for the ICD-9 assessment period in female subjects

	Migrants	Non-migrants				
	
Disorders (ICD-9)	(*n =*244)	(*n =*632)	χ^2^_CMH_	*p*	*p*_adj_	OR_MH_
Hyperkinetic disorders	5.7	11.2	5.28	0.0215	0.3230	0.5 (0.3–0.9)
Conduct disorders	13.1	11.6	0.15	n.s.	n.s.	1.1 (0.7–1.8)
Reactions to severe stress	18.9	11.2	7.88	0.0050	0.0949	1.8 (1.2–2.7)
Emotional disorders specific to childhood**	6.6	15.0	10.78	0.0010	0.0205	0.4 (0.2–0.7)
Other behavioral and emotional disorders	6.6	5.5	0.05	n.s.	n.s.	1.1 (0.6–2.1)
Pervasive developmental disorders	2.5	1.3	n.a.	n.a.	n.a.	n.a.
Eating disorders	11.5	19.0	5.31	0.0213	0.3400	0.6 (0.4–0.9)
Mixed disorders of conduct and emotion	4.1	4.3	0.01	n.s.	n.s.	0.9 (0.4–1.9)
Depressive episodes	5.7	2.2	6.32	0.0120	0.2034	2.7 (1.3–5.9)
Personality disorders	7.4	6.8	0.06	n.s.	n.s.	1.1 (0.6–2.0)
Disorders of social functioning	0.8	4.7	n.a.	n.a.	n.a.	n.a.
Tic disorders	1.6	1.4	n.a.	n.a.	n.a.	n.a.
Schizophrenia	2.5	2.7	0.05	n.s.	n.s.	1.0 (0.4–2.6)
Obsessive–compulsive disorders	2.5	1.6	0.28	n.s.	n.s.	1.5 (0.5–4.3)
Disorders due to cannabinoids	0.0	0.6	n.a.	n.a.	n.a.	n.a.
Phobic anxiety disorders	1.6	1.4	n.a.	n.a.	n.a.	n.a.
Somatoform disorders	8.2	2.1	14.79	0.0001	0.0025	4.0 (1.9–8.1)
Mild mental retardation	1.6	1.7	n.a.	n.a.	n.a.	n.a.
Dissociative disorders	4.1	2.4	0.95	n.s.	n.s.	1.6 (0.7–3.7)
Bipolar affective disorders	2.5	1.1	n.a.	n.a.	n.a.	n.a.
Schizoaffective disorders	1.6	0.5	n.a.	n.a.	n.a.	n.a.

Data are presented for the total sample for each group (migrants vs. non-migrants) for female subjects (*n =*number of patients,%). Statistical comparisons for each disorder were performed using the SES-adjusted chi-square (χ^2^) comparisons. Significant *p*-values underwent α-adjustment according to the Bonferroni–Holm procedure with adjusted *p*-values indicated by *p*_adj_, while the level of statistical significance remained at *p <*0.05 (n.a.=not applicable due to low cell frequencies, n.s.=not significant). Data are presented in descending order with respect to the diagnostic frequency of the respective disorders in the total sample. Shaded values represent adjusted and unadjusted significant findings that were also detected for the ICD-10 period of assessment. *Violation of the strata homogeneity prerequisite of the Cochran–Mantel–Haenszel procedure (see text for details).

** A significant relationship was also found after α-adjustment in females for the ICD-9 period of assessment.

**Table 4:** Comparison of disorders for the ICD-10 assessment period in male subjects

	Migrants	Non-migrants				
	
Disorders (ICD-10)	(*n =*412)	(*n =*1,789)	χ^2^_CMH_	*p*	*p*_adj_	OR_MH_
Hyperkinetic disorders**/***	37.9	57.2	48.8*	0.0000	0.0000	0.5 (0.4–0.6)
Conduct disorders	11.7	10.8	0.0	n.s.	n.s.	1.0 (0.7–1.5)
Reactions to severe stress**/***	15.1	6.2	32.1*	0.0000	0.0000	2.6 (1.9–3.6)
Emotional disorders specific to childhood	4.9	6.2	0.5	n.s.	n.s.	0.8 (0.5–1.3)
Other behavioral and emotional disorders	7.3	8.2	0.5	n.s.	n.s.	0.9 (0.6–1.3)
Pervasive developmental disorders	14.1	10.5	5.5	0.0186	0.2969	1.5 (1.1–2.0)
Eating disorders	1.5	0.4	4.2	0.0398	0.5175	3.6 (1.2–10.9)
Mixed disorders of conduct and emotion	3.9	5.5	1.5	n.s.	n.s.	0.7 (0.4–1.2)
Depressive episodes	4.4	1.6	11.1	0.0009	0.0162	2.8 (1.6–5.2)
Personality disorders	1.5	0.5	3.9	0.0469	0.5631	3.4 (1.2–10.2)
Disorders of social functioning	4.9	2.0	8.8	0.0030	0.0505	2.4 (1.4–4.3)
Tic disorders	5.8	3.6	4.4	0.0355	0.5322	1.7 (1.1–2.8)
Schizophrenia	2.9	1.3	3.5	n.s.	n.s.	2.1 (1.0–4.2)
Obsessive–compulsive disorders	1.0	1.8	n.a.	n.a.	n.a.	n.a.
Disorders due to cannabinoids	3.4	2.2	1.3	n.s.	n.s.	1.5 (0.8–2.9)
Phobic anxiety disorders	0.0	1.4	n.a.	n.a.	n.a.	n.a.
Somatoform disorders	0.0	0.3	n.a.	n.a.	n.a.	n.a.
Mild mental retardation	1.5	0.7	0.9	n.s.	n.s.	1.8 (0.7–4.8)
Dissociative disorders	0.0	0.2	n.a.	n.a.	n.a.	n.a.
Bipolar affective disorders	1.5	0.3	9.4	0.0022	0.0399	7.0 (2.0–24.5)
Schizoaffective disorders	1.0	0.3	n.a.	n.a.	n.a.	n.a.

Data are presented for the total sample for each group (migrants vs. non-migrants) for male subjects (*n =*number of patients,%). Statistical comparisons for each disorder were performed using the SES-adjusted chi-square (χ^2^) comparisons. Significant *p*-values underwent α-adjustment according to the Bonferroni–Holm procedure with adjusted *p*-values indicated by *p*_adj_, while the level of statistical significance remained at *p <*0.05 (n.a.=not applicable due to low cell frequencies, n.s.=not significant). Data are presented in descending order with respect to the diagnostic frequency of the respective disorders in the total sample. Shaded values represent adjusted and unadjusted significant findings that were also detected for the ICD-9 period of assessment.

* Violation of the strata homogeneity prerequisite of the Cochran–Mantel–Haenszel procedure (see text for details).

** Significant relationship also found after α-adjustment in females for the ICD-10 period of assessment.

*** A significant relationship was also found after α-adjustment in females for the ICD-10 period of assessment.

**Table 5:** Comparison of disorders for the ICD-10 assessment period in female subjects

	Migrants	Non-migrants				
	
Disorders (ICD-10)	(*n =*290)	(*n =*959)	χ^2^_CMH_	*p*	*p*_adj_	OR_MH_
Hyperkinetic disorders**/***	14.5	23.6	10.1*	0.0015	0.0276	0.6 (0.4–0.8)
Conduct disorders	13.8	12.9	0.03	n.s.	n.s.	1.1 (0.7–1.6)
Reactions to severe stress**/***	31.7	14.0	44.65	0.0000	0.0000	2.8 (2.1–3.8)
Emotional disorders specific to childhood	4.8	9.1	5.15	0.0233	0.3724	0.5 (0.3–0.9)
Other behavioral and emotional disorders	4.8	4.4	0.02	n.s.	n.s.	1.1 (0.6–2.0)
Pervasive developmental disorders	7.6	4.5	4.39	0.0361	0.5410	1.8 (1.1–3.1)
Eating disorders	7.6	13.3	5.44	0.0197	0.3346	0.6 (0.3–0.9)
Mixed disorders of conduct and emotion	5.5	7.5	1.18	n.s.	n.s.	0.7 (0.4–1.2)
Depressive episodes	6.9	9.2	1.08	n.s.	n.s.	0.7 (0.4–1.2)
Personality disorders	6.9	6.8	0.01	n.s.	n.s.	1.0 (0.6–1.7)
Disorders of social functioning	5.5	4.7	0.13	n.s.	n.s.	1.2 (0.6–2.1)
Tic disorders	0.7	0.9	n.a.	n.a.	n.a.	n.a.
Schizophrenia	3.4	2.1	1.36	n.s.	n.s.	1.7 (0.8–3.7)
Obsessive–compulsive disorders	0.7	2.6	n.a.	n.a.	n.a.	n.a.
Disorders due to cannabinoids	3.4	2.0	1.36	n.s.	n.s.	1.7 (0.8–3.8)
Phobic anxiety disorders	1.4	2.8	n.a.	n.a.	n.a.	n.a.
Somatoform disorders	0.7	1.6	n.a.	n.a.	n.a.	n.a.
Mild mental retardation	2.8	0.2	14.69	0.0001	0.0025	14.1 (2.9–68.5)
Dissociative disorders	2.8	1.0	3.51	n.s.	n.s.	2.7 (1.1–6.9)
Bipolar affective disorders	2.8	0.7	6.46	0.0110	0.1983	4.0 (1.4–11.3)
Schizoaffective disorders	2.1	0.5	4.07	0.0437	0.6111	3.8 (1.2–12.7)

Data are presented for the total sample for each group (migrants vs. non-migrants) for female subjects (*n =*number of patients,%). Statistical comparisons for each disorder were performed using the SES-adjusted chi-square (χ^2^) comparisons. Significant *p*-values underwent α-adjustment according to the Bonferroni–Holm procedure with adjusted *p*-values indicated by *p*_adj_, while the level of statistical significance remained at *p*<0.05 (n.a.=not applicable due to low cell frequencies, n.s.=not significant). Data are presented in descending order with respect to the diagnostic frequency of the respective disorders in the total sample. Shaded values represent adjusted and unadjusted significant findings that were also detected for the ICD-9 period of assessment.

* Violation of the strata homogeneity prerequisite of the Cochran–Mantel–Haenszel procedure (see text for details).

** Significant relationship also found after α-adjustment in males for the ICD-10 period of assessment.

*** A significant relationship was also found after α-adjustment in males for the ICD-10 period of assessment.

Schizophrenia was more frequently observed in male migrants than in non-migrants, but for the ICD-9 period only (Tables 2 and 4). There were higher rates of mild mental retardation according to the ICD-10 in female migrants compared to the rates observed in non-migrants. For ICD-9, this relationship was found in males, although at unadjusted levels only (*p*=0.045, χ^2^=4.023). The findings related to schizophrenia and mild mental retardation were not affected by SES.

Somatoform disorders were more frequently diagnosed in female migrants during the ICD-9 assessment period. However, no significant differences were found for either males or females during the ICD-10 period. Disorders of social functioning were found at higher rates in male non-migrants for ICD-10, but on pre-adjusted significance levels only. The complete results of the statistical comparisons for the ICD-9 assessment period are shown in Tables 2 and 3. There were no effects of SES on the diagnostic frequencies observed for the comparisons mentioned (somatoform disorders, disorders of social functioning).

### Disorders with higher diagnostic rates in non-migrants

There were higher diagnostic rates for hyperkinetic disorders among non-migrants during the ICD-10 assessment period than that among migrants of both genders. For ICD-9, these effects surpassed α-adjustment for males alone, but not for females (*p*=0.0215, χ^2^=5.283). These significant relationships are shown in Fig. 2. There were strong interaction effects for SES for all four comparisons on hyperkinetic disorders (ICD-10 vs. ICD-9, males vs. females). Emotional disorders with onset in childhood showed higher rates in female non-migrants compared to their migrant counterparts for ICD-9. For ICD-10, the adjusted significance levels were barely reached (*p*=0.0037, χ^2^=8.419). For males, this relationship was initially significant for ICD-9 but did not surpass significance upon correction (*p*=0.030, χ^2^=4.688). SES did not influence differences in the diagnostic rates of emotional disorders with onset in childhood.

## Discussion

This article provides an estimate of the rates of diagnosed mental ICD-9- and ICD-10-defined disorders among juvenile patients from a large population with or without a migration background who requested mental health services, as measured over the course of 20 years. One of the values of this study is its extension over 20 years with separate assessment periods of equal length that involved two subsequent classification systems, particularly because some of the diagnostic criteria changed during the transition from ICD-9 to ICD-10. In addition, both the sample size and the proportion of patients with a migration background in this study are considerably larger than those in previous studies (18, 20, 21, 38). A further advantage is that the effects of SES were controlled for, supporting previous studies in adults that suggested that differences in SES should be considered a crucial risk factor for specific mental health problems (39), particularly for psychotic disorders (40) and depression (41).

The most common disorder among both boys and girls were hyperkinetic disorders, conduct disorders, emotional disorders specific to childhood and reactions to severe stress and adjustment disorders. There were also higher rates of eating disorders in girls. Dissimilar diagnostic rates were found for a number of disorders that were dependent on both gender and migration status. Substantially higher diagnostic rates for patients with a migration background were found for reactions to severe stress and adjustment disorders among both girls and boys, for somatoform disorders among girls only and for bipolar disorders, depressive episodes, other behavioral and emotional disorders, and schizophrenia among boys. Of particular interest are the present results with respect to schizophrenia, a diagnosis that was more frequently observed in male migrants than in non-migrants (ICD-9 only), which was a finding similar to that noted in other studies. Indeed, a British population-based epidemiological survey found a higher risk for first-onset psychosis in black and minority ethnic subgroups, with a higher incidence found for first- and second-generation immigrants (42). However, there are indications that a substantial part of the increased risk is associated with socioeconomic disadvantage and social adversity (40). The finding that hyperkinetic disorders were less frequently observed in children with a migration background in our study is consistent with previous findings from Germany (43), with the major difference being that in these findings, we observed strong interactions with SES. This result is likely related to the fact that patients with a migration background in this sample had a significantly lower SES than non-migrants. Consequently, the effects found highlight the importance of focusing prevention strategies related to mental health problems on populations with low SES when trying to address patients and families with a migration background. Patients with a migration background were more likely to be outpatients than inpatients or day-care patients compared to non-migrants. However, the underlying reasons for this are unclear. One could speculate that patients with a migration background would not want to engage in day-care or inpatient care for cultural reasons. The level of impairment may also be a factor, as patients with more severe symptomatology often receive day-care or even inpatient treatment. However, such explanations are speculative and need to be addressed in future research.

Cultural differences in the parents’ interpretation and perception of normal vs. abnormal child behavior could also be a confounding factor. This factor may be an even more apparent bias than those supported by cultural differences in the perception and opinion of ‘what is normal?’ and ‘what is abnormal?’, as such differences may be the underlying reasons why families eventually decide to seek professional help and subsequently gain access to mental health care services. Moreover, these findings are merely descriptive, as further data on the underlying motivation of the families to seek access to mental health services is not available. For example, the child of a migrant who migrated voluntarily could have a different experience of integration than a child from a family who migrated under irregular conditions. Of note, refugees who obtained German citizenship are unaccounted for because our study was only able to use citizenship as criteria. Moreover, the documentation system that we used does not differentiate between first- and second-generation migrants or the country that was left (only data on citizenship were available; however, using citizenship as a proxy for migrant status is an established and standardized procedure [38]). The KJP-BADO system also does not provide information about the length of stay in the immigration country as well as in the country that was left. These are crucial limitations to the use of the KJP-BADO system and should be addressed in future revisions of this documentation approach. In addition, the assessment of potential obstacles that patients with a migration background experience is crucial and should also be included when the KJP-BADO system is revised. Some hospitals provide translators to allow patients with a migration background to overcome linguistic barriers. Providing such services is an essential strategy for improving healthcare for patients with a migration background and should be included in future recommendations about providing mental health services to this population. Given the cultural differences outlined above, one might argue on a behavioral level that the lower prevalence of hyperkinetic disorders in migrants compared to non-migrants may stem from changes in the parental perception of behavioral abnormalities in their children. However, when the physician in charge of the case knows that the patient visiting has a migration background, the diagnostic process can thus be affected. The factor of having a migration background can thus more easily be incorporated into and interpreted within a subjective model that belongs to a physician who handles a patient's adjustment problems. While the model may relate to the symptoms with which the patient presents, the physician is also aware that the patient has a migration background, which can have associated health risks. This subjective element, which could result in a strong bias, is difficult to overcome because it presumably transpires subconsciously. Alternatively, it may be helpful to maintain a critical awareness for such highly relevant factors when encountering patients and families with a migration background.

Of the various hypotheses seeking to explain changes in vulnerability to mental health problems in migrant populations, the migration-stress-hypothesis is of particular importance. This concept suggests that people with a migration background have an increased risk for emotional and behavioral disturbances due to a link with co-varying problems in psychosocial adaptation (44). Additionally, the gender hypothesis suggests an interaction between gender-specific vulnerabilities and characteristics that are specific for the sample. Finally, an origin-related hypothesis upholds the concept that different ethnicities may face an increased risk for a variety of problems in psychosocial adaptation. There is preliminary evidence that people with a migration background from southern Europe (e.g. Italy, Spain) and southeastern European countries (e.g. former Yugoslavia, Turkey) may face a more stressful process of acculturation (44), which should also be investigated in future studies.

These findings are subject to some limitations. First, although the definition of migration background by citizenship is an established procedure that has been implemented in previous studies (45, 46), there is no information provided about the duration of stay in the host country and the degree of acculturation within the individual patient. Moreover, people with multiple citizenships, although they represent only a minor proportion of people living in the studied catchment area, could produce a small but important bias. Second, the KJP-BADO form is designed to assess the country of origin based on the patient's citizenship. However, in this sample, many patients were classified based only on a non-citizenship-specific migration background. This is of concern because the specific ethnic background has been found to account for differences in prevalence rates of mental disorders in patients with a migration background (27, 31). Third, an important aspect of migration background appears to be the time of migration. A recently published longitudinal study in a representative sample of Swiss students revealed decreasing rates of internalizing problems in females with increased time from migration (47). These notions could not be addressed in this study but should be considered in future prospective studies.

The results of this study deserve further and careful consideration with regard to numerous points. The percentage of migrants in this sample, as assessed by the number of non-German-citizens, was of considerable size and was representative of the proportion of migrants living in the catchment area of the hospital. This finding suggests that members of this migrant population who were in need of treatment gained equal access to mental health services when compared to non-migrant patients. Additionally, one must note that the subjects involved in this study came from a rather densely populated area, prompting the question of whether similar findings would still be observed when studying more rural populations. This limitation also refers to the interactions found in this study between differences in diagnostic frequencies and SES for migrants and non-migrants, particularly because these differences may be more pronounced in more densely populated areas.

Given the relevance of migration in multi-cultural Europe and the current efforts being made at the European level to work toward more equitable health care systems, we hope that this work can contribute, even to a small extent, to a better understanding of the interaction between policy and healthcare. In particular, as outlined by the European Commission, the coherence and coordination between development policies and national migration and links with health policies should receive more attention and, in general, deserves increased efforts to be significantly supported (48). As suggested by the European Commission, the ‘sometimes overly sanguine debate on the “win-win potential” needs to be better balanced by taking the downsides of migration seriously, in particular its social costs and the risks of households becoming dependent on income from remittances’ (48). In view of the outlined downsides of migration and its associated risks, these issues might be particularly relevant for irregular migrants (undocumented migrants), children and adolescents, people requiring mental healthcare, and people with chronic conditions (49). As a consequence, young people with an irregular migration status and chronic mental health problems might be at a particular risk for worsened access to care. As outlined by the European Union Agency for Fundamental Rights (FRA), migrants in an irregular situation should have access to healthcare, and children with irregular migration status are thought to face legal and practical obstacles to accessing healthcare. Migrants in an irregular situation can present with psychosomatic problems, problems that can also be related to the use of drugs and alcohol and to posttraumatic stress disorders, psychotic disorders, and depressive symptoms (49, 50). In Germany, people with irregular migration status may not seek care for fear of deportation, though the doctor in charge of the case has an obligation to maintain doctor-patient confidentiality. A potential prevention strategy could relate to informing people about aspects of doctor-patient confidentiality, in particular with respect to migration status and possible irregular migration. However, because the data presented here are related to a more densely populated area in only one country, it is not clear whether identical findings would be detected in more rural areas, particularly with respect to the issue of migrants having equal access to mental health services. Thus, more studies on this topic are warranted.

## Conclusion

In summary, the results of this study provide preliminary evidence that the prevalence of various psychiatric disorders in children and adolescents is influenced by migration background and SES. With respect to research on migration and mental health, the KJP-BADO system is in need of significant revisions. The interaction of migration background and low SES may increase the risk for specific disorders, particularly in terms of reactions to severe stress and adjustment disorders in males.

## Disclosure

B.H.D. has served as a consultant to Eli Lilly and Co. and has received industry research funding from Medice and Vifor. M.H. has served in an advisory or consultancy role for Lilly, Novartis, Shire and Bristol-Myers Squibb and received conference attendance support or was paid for public speaking by AstraZeneca, Bristol-Myers Squibb, Janssen-Cilag, Lilly, Medice, Neuroconn, Novartis and Shire. He has received research support from the German Research Foundation (DFG) and the German Federal Ministry of Education and Research (BMBF). C.M.F. has been on the speaker's corner of Eli Lilly. L.W. has received research funding from the Danone Institute, which was not related to this study, and received conference attendance support or was paid for public speaking by AstraZeneca, Bristol-Myers Squibb, Janssen-Cilag, Salmon Pharma and Shire. F.P. was a member of the advisory boards of Lilly, Janssen Cilag, AstraZeneca and Novartis. F.D.Z. was the recipient of an unrestricted award donated by the American Psychiatric Association (APA), the American Psychiatric Institute for Research and Education (APIRE) and AstraZeneca (Young Minds in Psychiatry Award). He also received research support from the German Federal Ministry for Economics and Technology, the German Society for Social Pediatrics and Adolescent Medicine, the Paul and Ursula Klein Foundation, the Dr. August Scheidel Foundation, the IZKF fund of the University Hospital of RWTH Aachen University, and a travel stipend donated by the GlaxoSmithKline Foundation. He is the recipient of an unrestricted educational grant, travel support and speaker honoraria from Shire Pharmaceuticals, Germany. He also receives editorial fees from Co Action Publishing, Sweden. In addition, he has received support from the Raine Foundation for Medical Research (Raine Visiting Professorship).
